# Supplementary material for: The Metabolite Content of the Post-Culture Medium of the Tree Fern Cyathea delgadii Sternb. Cell Suspension Cultured in the Presence of 2,4-D and BAP
Source: Int J Mol Sci. 2022 Oct 4;23(19):11783. doi: 10.3390/ijms231911783 (PMC9569838; doi:10.3390/ijms231911783)
Supplement: Supplementary file 1 [file ijms-23-11783-s001.zip › ijms-1908331-supplementary.pdf]

Supplementary Table S1. List of identified compounds using GC-MS

Compound	RT [min]	RI	Height	Area	S/N	class	Number of compounds identified within the class
Glycerol	9,323742	1275,176	711811,7	1014213	26,61958	alcohol	4
R-(-)-1-Amino-2-propanol	10,13326	1332,386	1684700	2765975	103,3888	alcohol	
1,2,4-benzenetriol	13,5414	1595,33	44877960	78537440	28,72021	alcohol	
1-Hexadecanol	17,47334	1958,374	1089416	1918272	106,3345	alcohol	
Hydroxylamine	6,976822	1070,124	8702739	20609051	121,1292	amine	4
UDP-N-acetylglucosamine	16,08899	1823,317	8041777	16461500	5,146434	amine	
N-acetylmannosamine	18,94951	2112,248	90119,64	202925,2	8,447474	amine	
n-acetyl-d-hexosamine	19,08897	2127,163	6061407	10431642	46,85211	amine	
Alanine	7,191107	1091,812	19638151,5	36516334,8	1088,349	amino acid	10
Sarcosine	7,344167	1107,304	249412,3	342767,8	11,53388	amino acid	
Valine	8,50062	1217,005	4858378	13354560	164,9823	amino acid	
proline	9,660475	1298,974	90565880	164617000	2888,208	amino acid	
Glycine	9,7251	1303,541	29038300	81379789,2	1044,492	amino acid	
Threonine	10,83053	1381,663	2949845	5303479	24,73374	amino acid	
L-5-Oxoproline	12,57201	1518,089	776394400	1346671000	29121,3	amino acid	
L-Glutamic acid	13,76248	1614,443	4038458	6527331	301,1509	amino acid	
Phenylalanine	13,84752	1622,001	5814585	10077240	48,85922	amino acid	
Ornithine	15,27267	1748,682	8230961	15993660	297,9681	amino acid	
Heptanoic acid	7,854367	1158,944	5788162	20208540	266,4101	fatty acid	12
Octanoic acid	9,218302	1267,724	335725,4	522620,8	6,791001	fatty acid	
Nonanoric acid	10,53462	1360,75	8003666	12454460	48,21593	fatty acid	
Decanoic acid	11,80332	1456,838	2654851	3951318	12,18863	fatty acid	
Lauric acid	14,17064	1650,724	4003397	7031527	24,13218	fatty acid	
METHYL PALMITATE	17,14001	1925,854	4478071	9139183	27,38671	fatty acid	
Pentadecanoic acid	17,34068	1945,433	1257972	1776703	122,7868	fatty acid	
Palmitic acid	18,29306	2042,038	178203891	292565787	1074,098	fatty acid	

Linoleic acid	19,83386	2207,426	638783	1016335	2,932702	fatty acid	
Oleic acid	19,89169	2214,149	3402521	5984490	20,51014	fatty acid	
Stearic acid	20,12297	2241,043	77271503,5	132194705	464,6577	fatty acid	
Icosanoic acid	21,80663	2439,579	1207270	2359773	7,277335	fatty acid	
5,6-Dihydrouracil	7,45641	1118,665	103988000	164591700	112,5235	nucleic acid	5
Uracil	10,17748	1335,511	90710808,3	141093161	6108,116	nucleic acid	
Thymine	11,02441	1395,365	304492527	488884860	20854,74	nucleic acid	
2-Thiouracil	11,5244	1434,614	12174470	20818360	511,4374	nucleic acid	
Cytosine	12,58222	1518,902	9346211	14272720	911,3381	nucleic acid	
lactic acid	6,286352	1000,238	267047253	950958902	282,0074	organic acid	29
Glycolic acid	6,504037	1022,271	65473060,8	196068337	69,18887	organic acid	
Methylmalonic acid	8,7047	1231,428	3081531	4117324	3,334467	organic acid	
4-hydroxybutyric acid	8,80674	1238,639	128383500	208915400	138,9214	organic acid	
Benzoic acid	8,993814	1251,86	25600340	45685710	1133,228	organic acid	
Nicotinic acid	9,633264	1297,05	13642320	22111730	828,7513	organic acid	
Succinic acid(or anhydride)	9,884962	1314,838	46637390	76172120	50,46546	organic acid	
Methyl succinic acid	10,04142	1325,896	737979,3	1019418	6,198655	organic acid	
Glyceric acid	10,08224	1328,78	2723424	3240191	2,946967	organic acid	
Succinic acid	10,25911	1341,28	10661710	18603790	11,53684	organic acid	
pyrrole-2-carboxylic acid	10,37135	1349,212	29713540	48536050	2677,229	organic acid	
4-Methyl benzoic acid	10,40877	1351,856	4387237	7894057	93,4573	organic acid	
3-aminoisobutyric acid	10,97679	1391,999	29879630	77793450	1081,669	organic acid	
GABA	11,79651	1456,296	3915140	6923215	141,7316	organic acid	
L-(-)-Malic acid	12,15365	1484,753	4233440	7474750	4,580928	organic acid	
isothreonic acid	12,82372	1538,145	6161975	9476098	6,667758	organic acid	
5-hydroxymethyl-2-furoic acid	12,93596	1547,088	1548220	2208645	1,675301	organic acid	
3-Hydroxybenzoic acid	13,13664	1563,079	8503989	13717830	410,404	organic acid	
4-Hydroxybenzoic acid	13,87473	1624,42	42490940	70962300	1171,358	organic acid	
vanillic acid	15,40193	1760,171	3653387	5915464	361,7907	organic acid	
(-)-Shikimic acid	15,87811	1802,743	1140398	1893441	7,644417	organic acid	

Citric acid	15,96995	1811,702	9481352	23918050	302,3145	organic acid	
3,4-dihydroxybenzoic acid	15,99716	1814,357	13856070	24497440	295,1632	organic acid	
Myristic acid	16,3509	1848,868	21435,25	45230,6	3,743196	organic acid	
5-Keto-D-Gluconic acid	17,24205	1935,809	249622,1	407987,8	42,85948	organic acid	
galactonic acid	17,42912	1954,06	21086490	38666090	13,49456	organic acid	
isohexonic acid	17,78286	1988,572	4659,486	5407,36	3,987616	organic acid	
Gluconic acid	17,80667	1990,895	23177555,3	42024558,2	14,73119	organic acid	
lactobionic acid	24,5311	2793,437	1682548	2700757	4,702557	organic acid	
2-Hydroxypyridine	6,038055	975,1068	569600,6	1046204	26,39459	other	12
butyrolactam	7,527838	1125,895	7080447	20817440	225,8003	other	
4-Hydroxypyridine	7,66049	1139,321	4915400	9117093	276,6492	other	
SPIRO(3.5)SILANONANONE	7,847563	1158,255	44371790	76082680	2029,835	other	
1,1-DICHLORO-2-PHENYLETHENE	9,837343	1311,473	934813	1443840	18,90926	other	
3-Aminopropionitrile	10,38156	1349,933	14316730	22549770	558,6666	other	
erythronic acid lactone	10,63666	1367,962	8828366	19336960	5,649822	other	
1,3-dihydroxypyridine	10,81693	1380,702	6423153	11315090	578,7346	other	
hydroquinone	11,07203	1398,73	4757308	7701704	383,8413	other	
Nicotinamide	12,07882	1478,791	3810816	7305001	168,6902	other	
acetaminophen	13,90194	1626,839	10665380	19898960	180,9615	other	
beta-mannosylglycerate	19,55495	2177	734337,5	1205928	3,198426	other	
HYDROCINNAMIC ACID/4-hydroxycinnamic acid	11,85093	1460,632	136583,2	134433,4	7,195429	phenolics	6
CINNAMIC ACID	14,26588	1659,189	243876,8	456864,3	9,147424	phenolics	
2-Coumaric acid/2-hydroxycinnamic acid	16,29308	1843,227	169213,2	202307,2	7,148858	phenolics	
Ferulic acid/trans-4-Hydroxy-3-methoxycinnamic acid	18,72503	2088,238	1433168	2666955	365,1676	phenolics	
trans-4-Hydroxy-3-methoxycinnamate/trans-4-Hydroxy-3-methoxycinnamic acid	18,73523	2089,33	1615574	1823734	47,93757	phenolics	
Caffeic acid3,4-dihydroxycinnamic acid	19,14339	2132,983	3528194	6707794	59,08244	phenolics	
enolpyruvate	6,653695	1037,419	36378340	100385200	39,36433	phosphate	6
Pyrophosphoric acid	9,279525	1272,051	1085775000	1868572000	105979,2	phosphate	

phosphate	9,306735	1273,974	7050545	18687070	59,24483	phosphate	
glucose-1-phosphate	15,3305	1753,822	118259490	203274240	215,6438	phosphate	
glucose-6-phosphate	20,72501	2311,048	1076078	2167622	185,9737	phosphate	
inositol-4-monophosphate	21,54133	2406,417	252440,9	495150	29,27008	phosphate	
erythrose	11,70808	1449,249	2728829	4790982	2,952816	sugar	27
xylonolactone	13,65704	1605,07	55273,3	149167,6	6,37598	sugar	
D-(-)-Ribose	14,40534	1671,585	84093020	175114000	53,81636	sugar	
Lyxose	14,41894	1672,795	582777,4	939312,6	54,62738	sugar	
D-Xylulose	14,42574	1673,399	325700,9	610548,7	14,07167	sugar	
1,6-Anhydro-beta-D-glucose	14,71146	1698,796	124750200	229150700	79,83541	sugar	
methylhexose nist	15,8475	1799,778	1160765	1795277	4,918575	sugar	
D-(+)-Galactose	16,39511	1853,182	118890,3	172845,6	26,90545	sugar	
L-(-)-Sorbose	16,49375	1862,805	12779130	31050770	1512,61	sugar	
D-(-)-Fructose	16,52436	1865,792	1432237813	2362884445	549,0305	sugar	
Tagatose	16,54477	1867,783	9051,094	10026,69	2,669873	sugar	
glucose	16,67062	1880,061	258719854	416084441	162,7234	sugar	
glycerol-3-galactoside	20,02774	2229,969	2462719	4445967	10,72644	sugar	
glycerol-3-galactoside 2	20,66379	2303,929	113084,8	243058,5	3,473709	sugar	
Salicyl alcohol-beta-glucoside	22,38826	2512,283	559885,8	1083993	42,39275	sugar	
Sucrose	22,83724	2568,405	1906690666	7094500115	5445,98	sugar	
alpha-Lactose	23,58553	2666,515	4095469,13	6783524,23	3,269258	sugar	
Maltose	23,7522	2688,886	151428,5	290275,8	26,70021	sugar	
D-(+)-Trehalose	24,12634	2739,107	510663200	1902343000	4951,238	sugar	
Melibiose	24,24199	2754,629	610831,475	1246917,13	35,45736	sugar	
Turanose	24,57872	2799,829	1366022	1926392	3,817898	sugar	
beta-gentiobiose	24,67396	2812,612	1985346	4643381	8,64723	sugar	
Trehalose	25,25899	2891,139	668445,8	1537452	50,61255	sugar	
inulotriose	30,47323	3448,303	137233,2	294766,6	10,39085	sugar	
1-kestose	30,58888	3460,008	60261,38	116125,1	15,53266	sugar	
Maltotriose	31,11949	3513,713	1569942,9	9151420	94,00894	sugar	

D-Panose	32,77594	3681,37	742836,948	4211181,62	3,219192	sugar	
Meso erythritol	12,38154	1502,912	25239026	54273787	108,3589	sugar alcohol	15
Pyrogallol	12,80671	1536,79	26689010	44466440	2153,391	sugar alcohol	
deoxypentitol	13,70466	1609,303	9344517	17365170	5,980139	sugar alcohol	
Xylitol	14,22506	1655,561	303835,3	655800,3	6,23469	sugar alcohol	
levoglucosan	14,48016	1678,237	1119476	1761105	3,128825	sugar alcohol	
D-Arabitol	14,89172	1714,82	3631846	5506616	10,15065	sugar alcohol	
Ribitol	14,94615	1719,657	2181283,9	3323971	16,25092	sugar alcohol	
pinitol	16,44273	1857,828	1156759	1793938	7,128245	sugar alcohol	
L-Iditol	16,97674	1909,926	366103923	590457538	2447,581	sugar alcohol	
D-(-)-Sorbitol	17,1196	1923,863	4716104	6875739	3,018129	sugar alcohol	
Myo-Inositol	18,69782	2085,328	119257600	204229900	76,32036	sugar alcohol	
glucoheptulose	21,1944	2365,627	85052880	149287000	54,43064	sugar alcohol	
lactitol	24,2692	2758,282	12305390	22372090	53,59646	sugar alcohol	
maltitol	24,7862	2827,678	2375626	6438989	6,639638	sugar alcohol	
Galactinol	25,14675	2876,073	31007210,1	79895521,6	113,5515	sugar alcohol	
